# Changes of Heart Rate and Lipid Composition in Mytilus Edulis and Modiolus Modiolus Caused by Crude Oil Pollution and Low Salinity Effects

**DOI:** 10.3390/jox11020004

**Published:** 2021-05-14

**Authors:** Igor Bakhmet, Natalia Fokina, Tatiana Ruokolainen

**Affiliations:** Karelian Research Center of RAS, Institute of Biology, Pushkinskaya St., 11, 185910 Petrozavodsk, Russia; igor.bakhmet@gmail.com (I.B.); truok@krc.karelia.ru (T.R.)

**Keywords:** oil pollution, cardiac activity, phospholipids, cholesterol, triacylglycerols, mussel

## Abstract

Blue mussels, *Mytilus edulis*, inhabiting tidal zones, are naturally exposed to fluctuating environmental conditions (e.g., fluctuations in temperature and salinities), while horse mussels, *Modiolus modiolus*, live under relatively invariable shelf water conditions. The present investigation tested the hypothesis: blue mussels, in comparison to horse mussels, have an increased ability to tolerate the stress of pollution combined with low salinity. To assess the response of blue mussels and horse mussels to oil pollution at seawater salinities of 25 psu (normal) and 15 psu (low), we used a combination of heart rate and lipid composition as physiological and biochemical indicators, respectively. A sharp decrease in heart rate as well as important fluctuations in cardiac activity was observed under all oil concentrations. Modifications in the concentrations of the main membrane lipid classes (phosphatidylcholine, phosphatidylethanolamine, and cholesterol) and storage lipids (primarily triacylglycerols) in response to different crude oil concentrations were time- and dose-dependent. Both chosen indicators showed a high sensitivity to crude oil contamination. Furthermore, both bivalve species showed similar responses to oil pollution, suggesting a universal mechanism for biochemical adaptation to crude oil pollution.

## 1. Introduction

The oil and gas industry in the northern latitudes constantly pushes the boundaries of resource exploration northward. In this context of growing human pressure on an ecosystem considered fragile and pristine, there is a need to study the basic biology of Arctic key species and their sensitivities to human impact combined with environmental variability. Coastal and shelf areas are considered the most valuable, but at the same time, the most sensitive parts of the Arctic have high biodiversity, unique ecology, and economic value [1,2]. Blue mussels (*Mytilus edulis* L.) and horse mussels (*Modiolus modiolus* L.) are considered ideal sentinel species in coastal and shelf water ecosystems [3,4,5]. These bivalves possess both defense mechanisms (behavioral, physiological, etc.) and acclimation capacities that allow them to develop a high tolerance to stress. Blue mussels are the dominant bivalve species in the White Sea coastal ecosystems, while the horse mussel, living at depths of 5–20 m with no exposure to tidal effects, is a typical species in the White Sea subtidal zone.

To assess the response of blue mussels and horse mussels to crude oil pollution, we suggested using a physiological (heart rate (HR)) indicator and a biochemical (lipid composition) indicator. A significant correlation between mussel HR changes and a variation in environmental factors was shown in previous studies [6,7]. Moreover, the high sensitivity of mussel HR to some pollutants such as trace metals, ammonia, and diesel oil has been well established [8,9]. Crude oil is known to have a nonpolar narcotic effect on organisms because of the accumulation of pollutants in lipid-rich tissues [10]. Crude oil compounds, primarily polycyclic aromatic hydrocarbons (PAHs), modify membrane lipids through the activation of lipid peroxidation reactions, thereby altering the properties of the membranes including fluidity and permeability [11,12]. Moreover, the exposure of marine mussels to petroleum products can affect the biosynthesis of phospholipids and triacylglycerols (TAG), and this is reflected in the structure and function of biological membranes [12,13,14]. Thus, the assessment of HR and lipid composition including membrane lipids (cholesterol and various fractions of phospholipids) and storage lipids (mainly triacylglycerols) in blue mussels and horse mussels will reveal the adaptive mechanisms of the sensitive bivalves under the varying environmental conditions of coastal and shelf waters.

Thus, the goal of the present investigation was, first, to test the hypothesis that organisms such as blue mussels, which are naturally exposed to a fluctuating coastal environment, have an increased ability to tolerate pollution stress compared to horse mussels living under relatively invariable shelf water conditions. Second, we determined the sensitivity of blue mussels to crude oil exposure combined with low salinity conditions. Under low salinity conditions, alterations of some biochemical pathways may lead to an increase in the susceptibility of mussels to additional stress from heavy metal and diesel oil pollution [15,16].

## 2. Materials and Methods

### 2.1. Mollusc Sampling, Maintenance, and Acclimation

Experiments were carried out at the White Sea Biological Station (Chupa Inlet, Gulf of Kandalaksha, White Sea) of the Zoological Institute of the Russian Academy of Sciences. Blue mussels were collected in the Kruglaya Bay at a depth of 2.0 m from artificial substrates used for mussel rearing. Horse mussels were collected by scuba diving at a depth of 5.0–15.0 m. After sorting the animals by size and age, retaining those within 45–55 mm for blue mussels and 65–75 mm for horse mussels and cleaning encrusting organisms off the shells, the animals were divided among 12 Plexiglass aquaria containing 25 organisms each and acclimated to laboratory conditions for seven days with aerated seawater and constant light at a temperature of 10 °C. The 12 aquariums of 20 L comprised two groups of four aquaria with blue mussels acclimated to seawater with a salinity of 25 psu and 15 psu, respectively. The remaining group of four aquaria with a salinity of 25 psu contained horse mussels. Some of the aquarium water (3/4 of the volume) was replaced daily. Animals were kept without being fed to avoid specific dynamic action. One day prior to the experiment, optical sensors (CNY-70) were glued onto the animals’ shells to measure the HR.

### 2.2. Crude Oil Exposure Experiment

**Figure 1:**
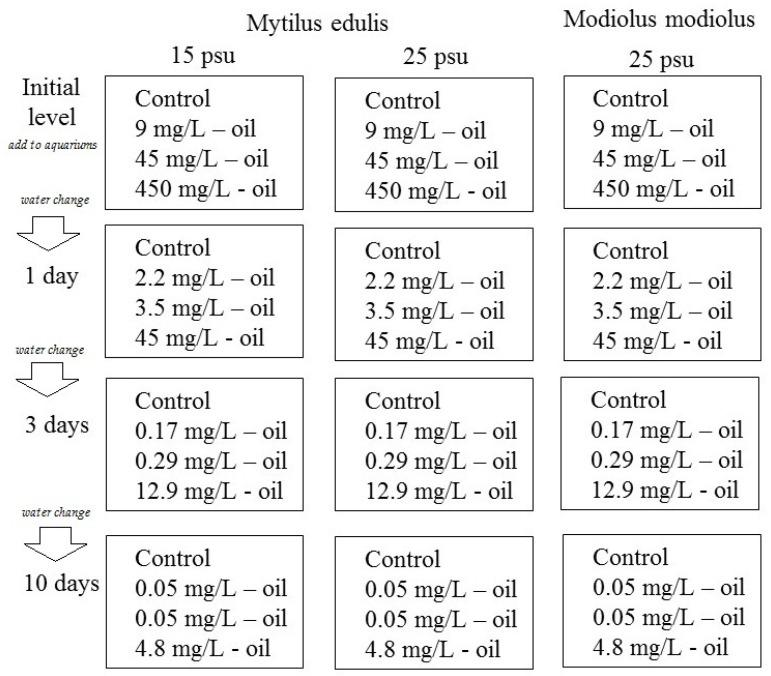
A scheme of the experimental design.

The experiment was a semi-static exposure setup with the goal of simulating an oil spill in a coastal environment, that is, with a decrease in the crude oil concentration over time. Twelve 15 L aquaria were filled with gravel (particle size 3–5 mm), a typical type of sediment in many littoral places in the White Sea [17]. A crude oil-in-water mixture was generated by mixing 100 mL crude oil (West-Surgut oilfield) in 900 mL seawater and shaking by hand for 10 min. The oil-in-water mixture was added to 12 aquaria in different amounts (0, 1, 5, and 50 mL) to generate a control, low, medium, and high crude oil exposure concentrations (in each of three aquaria). Finally, 10 L sea water of different salinities (15 or 25 psu) was added to each of the 12 aquaria with continuous mixing, generating final concentrations of 0 (control group), 9, 45, and 450 mg crude oil/L. The aquarium design with exposure organisms, seawater salinities, and initial nominal oil concentrations is summarized in Figure 1 and Table 1. Every 24 h during the experimental period, the water in the experimental aquaria was completely exchanged. It should be noted that the oil in the aquaria partly evaporated and was adsorbed to the tank walls and gravels.

After 1, 2, 3, and 10 days of exposure and before the exchange of the exposure water, water samples (200 mL) were taken from the middle layer of each aquarium to determine the total hydrocarbon content (THC) in the water column. Because the constant aeration of the water allowed for the mixing of all the aquarium layers, we therefore supposed that the concentration of THC was approximately equal in all the layers. To preserve the water samples until a chemical analysis could be performed, 10 mL of toluol was added. Excitation-photometry with column chromatography on Al_2_O_3_, followed by spectroscopy, was used (standard procedural instructions RD 52.24.476-95) for the analysis of THC in the aquarium water.

The mussels (*n* = 5 per treatment) were dissected before exposure start (control, day 0) and following 1, 3, and 10 days of exposure. Gills and digestive glands were taken from the mussels and stored in 97% ethanol at +4 °C until a lipid composition analysis could be performed.

### 2.3. Heart Rate Monitoring and Data Processing

The HR of the test organisms was recorded every 2 h for one day before exposure start and then daily using remote monitoring of the cardiac muscle volume (plethysmogram) based on infra-red radiation of the heart region and reflected light recording [18]. Optical sensors CNY-70 were used. A specially developed amplifier with a system of filters was employed, and a portable digital oscillograph (Fluke 125) transmitted the signal to a personal computer to be recorded as consecutive HR waves and processed using the FlukeView 3.0 software [7,19]. The time of a single heart contraction, the number of contractions per minute, and the standard error were calculated; then the 24 h average was determined.

### 2.4. Biochemical Analyses

The analyses were carried out using the facilities of the Equipment Sharing Center of the Karelian Research Center RAS.

Total lipids were extracted by the Folch method [20] using a chloroform:methanol mixture (2:1, volume ratio). Lipids were spotted onto Merck thin-layer chromatography plates (Darmstadt, Germany) and separated into different fractions of lipid classes: phospholipids, cholesterol, TAG, and cholesterol ethers, using petroleum ether:diethyl ether:acetic acid (90:10:1, volume ratio) as the mobile phase. The content of the phospholipids, TAG, and cholesterol esters was determined by the method suggested by Sidorov et al. [21] and the cholesterol level by the method of Endelbrecht [22]. The composition of the individual phospholipid fractions was determined by high performance liquid chromatography using the method of Arduini et al. [23] on a Nucleosil 100-7 column with the liquid phase acetonitrile:hexane:methanol:phosphorus acid (918:30:30:17.5, volume ratio) and an UV-spectrophotometer with 206 nm wave length. The position of the fractions on the TLC plates and HPLC chromatograms was determined using the following standards: phospholipid mixture (Supelco, Bellefonte, PA, USA), cholesterol (Sigma Aldrich, St. Louis, MO, USA), glyceryl trioleate (Sigma Aldrich, USA), and cholesteryl palmitate (Sigma Aldrich, USA).

The results of the lipid classes’ content in gills and digestive glands of blue mussels have been described earlier in our articles [24,25] and are not included in this paper.

### 2.5. Statistical Analysis

A statistical analysis was carried out using Stat Soft Statistica v7.0. Kolmogorov–Smirnov and Lilliefors tests were used to examine the normality of the distribution of the investigated lipid parameters. Because in the case of the lipid results the distribution deviated from normal, the significance of the differences in the mussels’ lipid composition in the experiment was estimated by the non-parametric Mann–Whitney U test and the Kruskall–Wallis test with post-hoc analysis by the Tukey–HSD test. The differences were considered significant at *p* < 0.05. Analysis of variance (two-ways ANOVA) and the control Dunnett’s test were used to estimate significance in heart activity with regard to different species’ reactions to pollution. Separate ANOVAs were performed to test the effects of low salinity on the mussels’ HR. A significance level of *p* < 0.05 was used in all statistical tests.

## 3. Results

### 3.1. Total Hydrocarbon Content (THC) in Aquarium Water

The initial nominal concentration of THC in the test aquaria was 9.0, 45.0, and 450.0 mg/L in the low, medium, and high treatments, respectively. As a result of the daily dilution by adding uncontaminated water to simulate a tidal effect, there was a significant reduction in the THC in the aquaria with the experimental mussels (Table 1).

### 3.2. Physiological Indices

**Figure 2:**
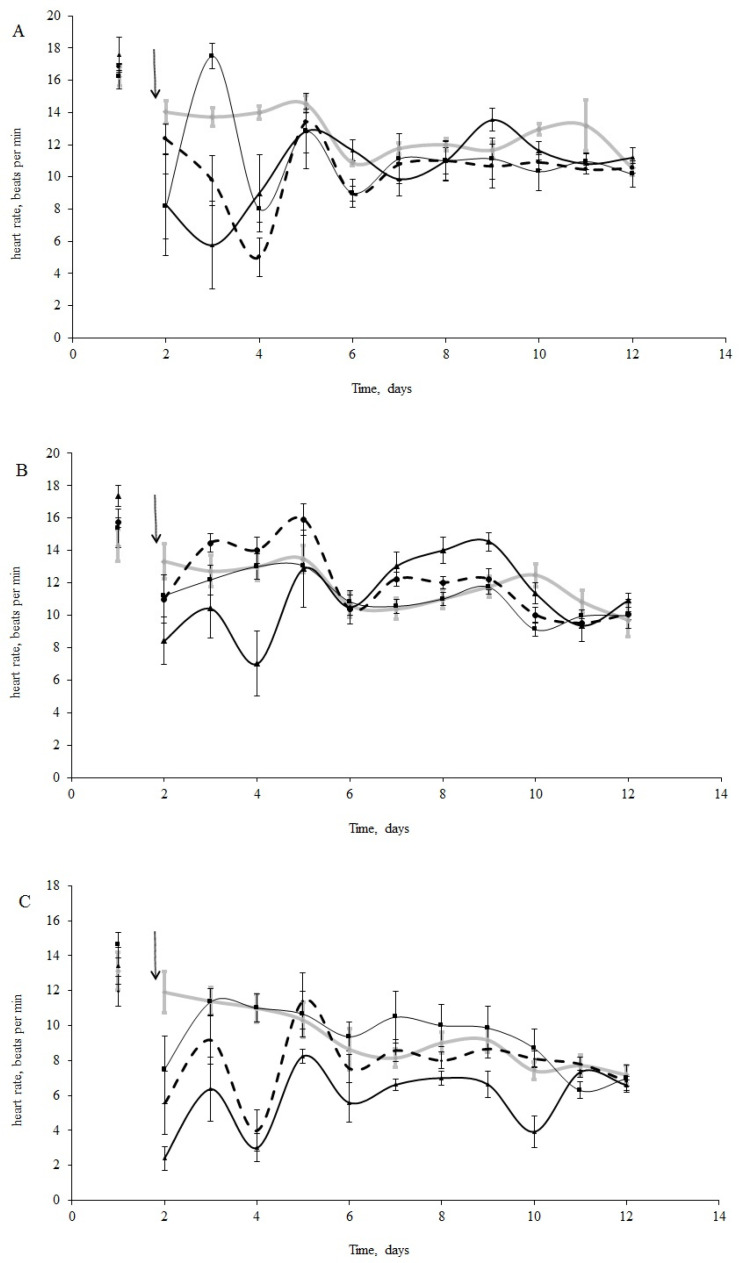
(**A**) Heart rate change in blue mussels under 25 psu salinity; (**B**) Heart rate change in blue mussels under 15 psu; (**C**) Heart rate change in horse mussels under 25 psu salinity (with no oil (grey line) and under exposure to low (L), medium (M), and high (H) crude oil concentrations: thin black line, thick black line, and dotted black line, respectively; the arrow points to the time of pollution water added). Each point represents the mean of seven individuals; error bars are a standard error.

The initial HR of blue mussels (*n* = 15 per treatment) varied from 14 to 18 bpm (16.2 ± 2.1 bpm) under normal salinity conditions (25 psu, Figure 2A) and from 9 to 19 bpm (14.3 ± 1.3 bpm) under low salinity (15 psu, Figure 2B) conditions before oil exposure. Horse mussels showed an HR between 10 and 14 bpm (12.6 ±1.2 bpm) (Figure 2C). It is worth mentioning that the HR of the molluscs in the control groups decreased, but not significantly, over the 11 days of the experiment (Figure 2A–C). Furthermore, bradycardia was observed in all experimental groups (under all experimental concentrations (Figure 2A–C). Cardiac activity oscillated for both species in the first days following the oil contamination in all treatments. These fluctuations were particularly noticeable in the horse and blue mussels under normal salinity (25 psu) (Figure 2A–C).

There were significant differences between species in their responses to crude oil exposure. There were also significant differences in the blue mussels between salinity treatments. According to the ANOVA analyses, we had significant influences of the species, crude oil concentrations, and the interaction of these factors during the first four days following the oil exposure. After that, the interaction became insignificant (Table 2). From the sixth day, only differences in HR between species were shown (Table 2). Furthermore, when comparing the HR changes under crude oil exposure in the blue mussels from the ‘low salinity’ and ‘normal salinity’ groups, the effect of the pollution disappeared after three days (Table 3).

The most prominent decrease in HR took place with the medium crude oil treatment for the horse mussels (Figure 2C) and in the low and high crude oil treatments for the blue mussels (Figure 2B). Thus, clear dose-dependent responses were not observed. However, abrupt cardiac arrest was observed in some of the horse mussels in the medium and high crude oil treatments, while in some of the blue mussels, the heartbeat halted only under high crude oil exposure. Another difference between the two species was the HR changes over the course of the experiment. The cardiac activity in the blue mussels in all the oil treatments returned to the control levels on the fourth day of exposure (Figure 2A,B), while the HR in the horse mussels became equal to the control gradually on the second, fourth, and tenth days in the low, medium, and high treatments, respectively (Figure 2C). In addition, the dependence of HR change on oil concentration was shown for the horse mussels (lgHR = −0.27 × lgC + 0.40; R^2^ = 0.97; *p* < 0.05). Interestingly, for the blue mussels (both salinity groups), a 10-fold increase in variance was shown only under a high oil concentration, followed by a return to former levels in 3–4 days. In contrast, the variances of HR in the horse mussels fluctuated significantly from the control values under all crude oil concentrations during nine days of the experiment under all crude oil concentrations.

When comparing the HR of the blue mussels between salinity conditions (Figure 2A,B), the HR of the mussels exposed to low salinity returned to the initial levels within two days under low and medium crude oil concentrations, while the HR in the normal salinity group was restored four days after the addition of oil under all concentrations (Figure 2A,C).

### 3.3. Lipid Composition

#### 3.3.1. Effects of Crude Oil Pollution on Horse Mussels

**Figure 3:**
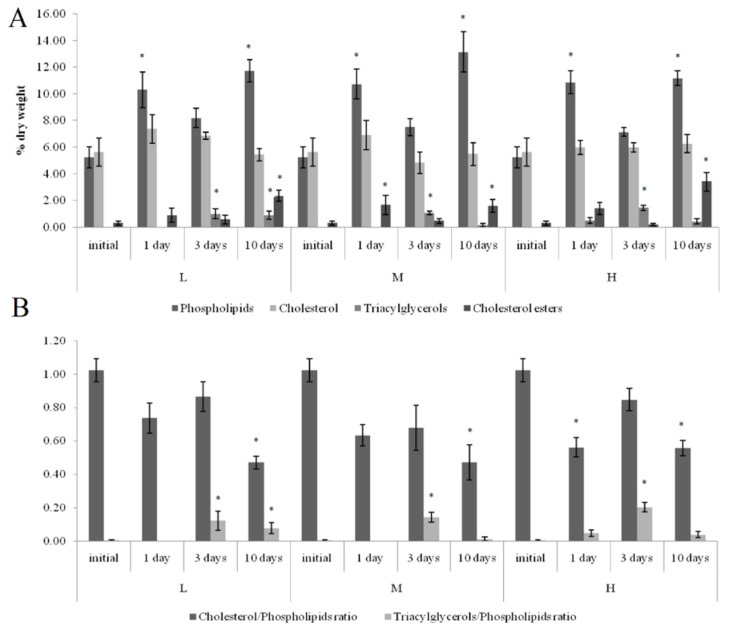
Lipid classes levels (**A**) and ratios (**B**) in the gills of horse mussels under low (L), medium (M), and high (H) crude oil concentrations. Values are means ± standard error (*n* = 5). * significant results in comparison with initial level; 1, 3—significant results in comparison with the first and third days of exposure. Differences were estimated by the nonparametric Kruskal–Wallis test, *p* < 0.05.

**Figure 4:**
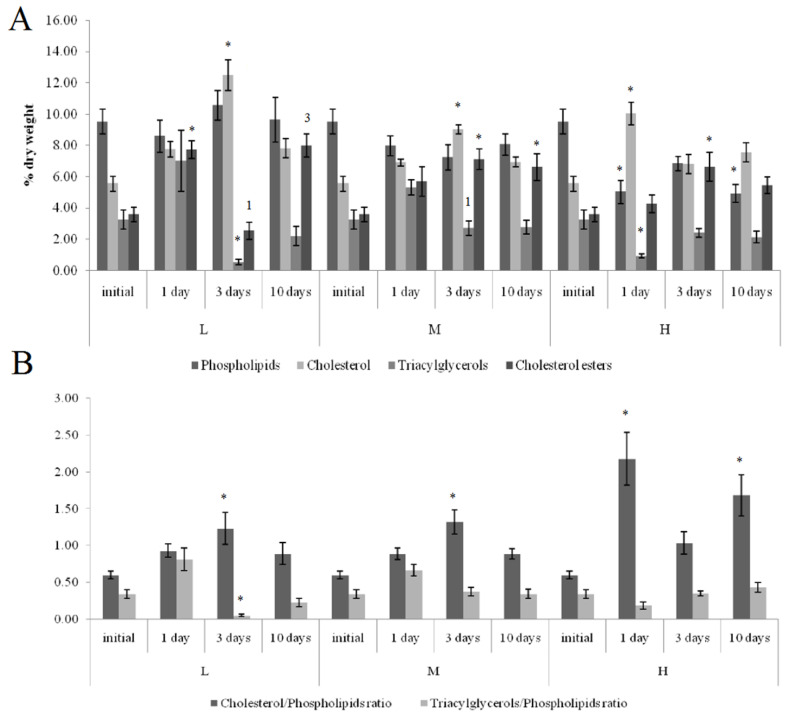
Lipid classes levels (**A**) and ratios (**B**) in the digestive glands of horse mussels under low (L), medium (M), and high (H) crude oil concentrations. Values are means ± standard error (*n* = 5). * significant results in comparison with initial level; 1, 3—significant results in comparison with first and third days of exposure. Differences were estimated by the nonparametric Kruskal–Wallis test, *p* < 0.05.

On the first and tenth days of the experiment, the PL content in the horse mussels’ gills was elevated in all crude oil treatments, while on the tenth day, the Ch/PL ratio decreased and the ECh concentration increased. In addition, on the third experimental day, the TAG levels and the TAG/PL ratio in the horse mussels’ gills increased (Figure 3). It should be noted that the Ch content in the horse mussel gills did not change during the experiment. An increase in the Ch/PL ratio against the lowered TAG content in the digestive glands of the horse mussels was observed on the third experimental day under exposure to the low and medium crude oil concentrations as well as on the first and tenth days under exposure to the high crude oil concentration (Figure 4). There was an increase in the ECh level in the digestive gland on the first and tenth days of exposure to the low crude oil concentration, and on the third and tenth days of exposure to the medium crude oil concentration (Figure 4).

**Figure 5:**
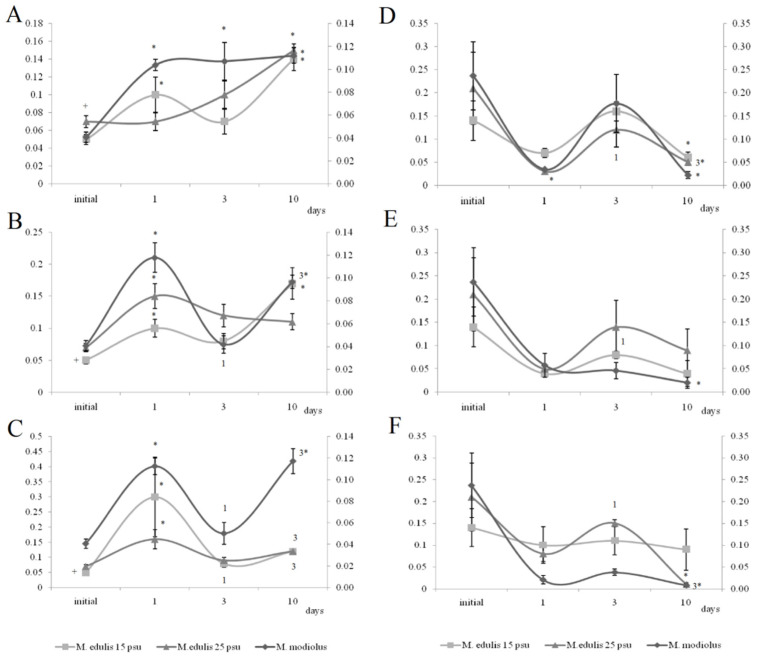
PEA/PC ratio in gills (**A**–**C**) and digestive glands (**D**–**F**) of horse mussels and blue mussels under low (L), medium (M), and high (H) crude oil concentrations. Values are means ± standard error (*n* = 5). * significant results in comparison with initial level; 1, 3—significant results in comparison with first and third days of exposure. Differences were estimated by the nonparametric Kruskal–Wallis test, *p* < 0.05 + significant differences between mussels acclimated to 15 and 25 psu were estimated by the nonparametric Mann–Whitney U test, *p* < 0.05.

Under exposure to the low crude oil concentration, the PEA/PC ratio in the gills of the horse mussels increased (Figure 5) on the first and tenth day. The PEA/PC ratio increased for horse mussels under exposure to medium and high crude oil concentrations on the first experimental day and declined on the third day. It then increased on the tenth day in all studied mussel groups. On the tenth day of the experiment, the PEA/PC ratio in digestive glands was shown to decrease under the low and high oil concentrations in horse mussels.

**Figure 6:**
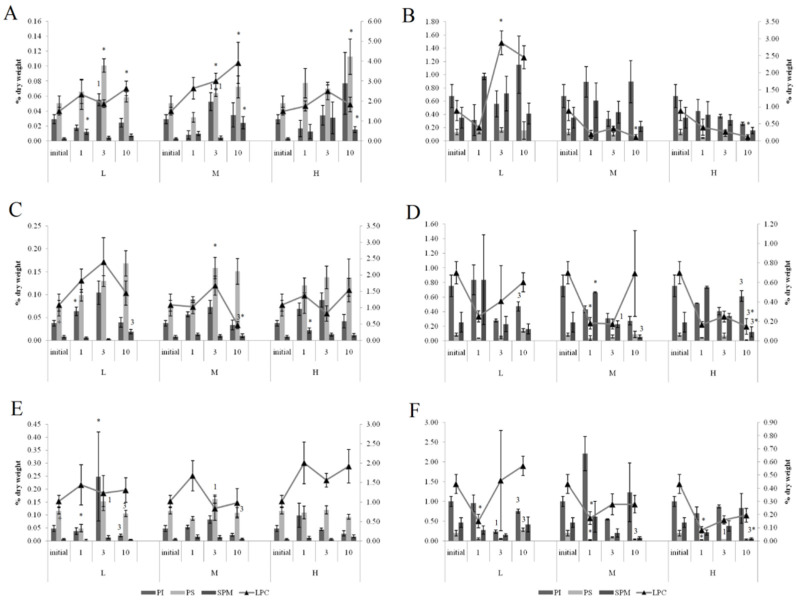
The content of some minor phospholipids and lysophosphatidylcholine (LPC) in gills (**A**,**C**,**E**) and digestive glands (**B**,**D**,**F**) of horse mussels (**A**,**B**) and blue mussels (**C**,**D**) low salinity; (**E**,**F**) normal salinity) under low (L), medium (M), and high (H) crude oil concentrations. Values are means ± standard error (*n* = 5). * significant results in comparison with initial level; 1, 3—significant results in comparison with first and third days of exposure. Differences were estimated by the nonparametric Kruskal–Wallis test, *p* < 0.05 + significant differences between mussels acclimated to 15 and 25 psu were estimated by the nonparametric Mann–Whitney U test, *p* < 0.05.

In the gills of the horse mussels, primarily on days 1 and 3 of the experiment with the low and medium oil concentrations, we observed elevated PI and PS levels (Figure 6), which declined by the end of the experiment (day 10). The SM content in the horse mussel gills increased on the first day (under the low oil concentration) and on the tenth day (under the medium and high oil concentrations) of the experiment. An increase in the LPC content was noted in the horse mussel gills on the tenth day of exposure to the low and medium oil concentrations. In the horse mussel digestive glands, the LPC level increased on the third day of exposure to the low crude oil concentration and decreased on the tenth day under exposure to the medium and high oil concentrations.

#### 3.3.2. Effects of Oil Pollution on Blue Mussels (Normal Salinity)

At both normal (25 psu) and low (15 psu) salinities in the blue mussels, similar changes in the level of the dominant membrane phospholipids PC and PEA were observed in response to all crude oil concentrations. Under exposure to the low crude oil concentration, the PEA/PC ratio in the gills of the blue mussels increased (Figure 5) on the tenth day. The PEA/PC ratio increased in mussel gills under exposure to medium and high crude oil concentrations on the first experimental day. It then increased on the tenth day in mussels, except for the medium crude oil concentration. In the digestive glands of the blue mussels, the PEA/PC ratio decreased under exposure to the low crude oil concentration on the first experimental day. The ratio increased in the blue mussels under exposure to the low and high crude oil concentrations. On the tenth day of the experiment, the PEA/PC ratio was shown to decrease under the low and high oil concentrations in all studied molluscs.

In the gills of the blue mussels, primarily on days 1 and 3 of the experiment with the low and medium oil concentrations, we observed elevated PI and PS levels (Figure 6), which declined by the end of the experiment (day 10). In the mussels exposed to the low crude oil concentration, the PI level decreased on the third experimental day and increased, together with the PS, on the tenth day. Under exposure to the medium crude oil concentration, the PI content increased and the PS content decreased on the first experimental day, whereas on the tenth day, only the PS level remained reduced. In the mussels, the PS level decreased on the first day of high crude oil concentration exposure and then rose on the third day; the SM content decreased on the tenth day.

#### 3.3.3. Effects of Low Salinity and Oil Pollution on Blue Mussels

The effect of the low salinity (15 psu) on the blue mussels reduced levels of PS, PEA, PC, and its lyso form (LPC) in the gills (Figure 5 and Figure 6). In the mussels acclimated to 15 psu salinity, increased PEA/PC ratio in the gills was noted on the first experimental under exposure to low, medium, and high crude oil concentrations. It declined on the third day in mussels acclimated to 15 psu under exposure to the high crude oil concentration and subsequently increased on the tenth day. In the digestive glands, the ratio increased in the mussels under exposure to the medium crude oil concentration on the third day. On the tenth day of the experiment, the PEA/PC ratio was shown to decrease under the low and high oil concentrations in all studied molluscs, except for the mussels under the high oil concentration.

Primarily on days 1 and 3 of the experiment with the low and medium oil concentrations, we observed elevated PI and PS levels in gills, which declined by the end of the experiment (day 10). In the mussels, an increase in the SM content was observed after one day of high oil concentration exposure. A decrease in the LPC level was noted in the gills of the mussels on the tenth day of medium concentration crude oil exposure. In the digestive glands of the mussels, the PI level increased on the tenth day of exposure to the low and high crude oil concentrations. At the same time, the PS and LPC content decreased under exposure to the high crude oil concentration.

## 4. Discussion

The THC in all treatments decreased drastically and by approximately one order of magnitude on the first day of the experiment because of the complete exchange of treatment water after 24 h of exposure. It is evident that 10 days was not enough to clean up the contamination. The next consequence of the fast change in oil concentration was the relatively short time of the mussels’ acclimation to the contamination.

### 4.1. Heart Rates

The higher HR of the blue mussels compared to that of the horse mussels suggests that the former has a higher level of metabolism. Blue mussels were demonstrated to have a significantly high oxygen consumption compared to horse mussels [3,26]. The observed bradycardia, common to all crude oil groups, reflects the overall depression of animal functional activity and, consequently, a lower rate of basic metabolism. Similarly, a relatively recent study recorded a decrease in the heart rate and basic level of metabolism in crab *Carcinus maenas* exposed to chronic pollution [27]. In our study, this response may be explained by several mechanisms. First, in the presence of pollution, bivalves have been shown to respond defensively by closing their valves and, consequently, stopping their filtration activity. Second, the work of Kuwasawa and Hill [28] provides direct electrophysiological evidence for serotonergic excitatory junctional potentials in the heart of *Mercenaria mercenaria*.

The fluctuation of the mussels’ HR during the first two to five days after crude oil exposure suggests an active acclimation to the oil pollution in a second phase [29]. It is known that the adaptation process is oscillatory by nature because of the inertia of adaptation mechanisms [30]. A similar observation was made in relation to the effect of iron and lipophilic contamination on mussels [31,32]. Our present study further suggests that, in general, mussels’ primary acclimation to pollution lasts for about four days, as observed by the recovery of the initial level of the mussels’ cardiac activity in that period. Nevertheless, two exceptions to this acclimation time were observed. First, the horse mussels under the high oil concentration required significantly more time for adaptation to the pollution, while the blue mussels from the ‘low salinity’ group under the low and medium oil concentrations returned to the control HR levels sooner—within two days. This suggests that the adaptation mechanisms of horse mussels are not as effective as those in blue mussels, further supporting our hypothesis based on the different habitats of these two species, which was quite stable for horse mussels (6–15 m depth) and extremely changeable for blue mussels. It is more difficult to explain the shorter time needed for adaptation in blue mussels under low salinity. One of the most plausible assumptions may be ‘pre-adaptation’ [30]. This group of molluscs faced a dramatic change in ambient sea water conditions 10 days before crude oil exposure, and the adaptation mechanisms in the blue mussels were therefore the most effective at that moment [30].

### 4.2. Lipid Composition

Lipids play an important role not only in an organism’s adaptation to new environmental conditions [33], but also in the accumulation of lipophilic contaminants including petroleum hydrocarbons. It was shown that tissues containing an increased level of membrane lipids, in particular phospholipids, accumulate oil products more rapidly than tissues with high levels of storage lipids (mainly TAG) [10,34]. Phospholipids and cholesterol are the main components of biological membranes and are exposed to environmental factors. Pollution-triggered activation of lipid peroxidation processes causes membrane phospholipid modifications that lead to a disturbance of the biological membrane integrity and permeability [13,14]. Our earlier studies of the modifications of the lipid and fatty acid composition in blue mussels in response to different concentrations of cadmium and copper ions as well as diesel fuel in seawater revealed the possibility of using some lipids and fatty acids as biomarkers to demonstrate the adverse effect of pollutants [24,25,29].

Horse mussels typically inhabit the subtidal zone and have no direct exposure to tidal cycles. At the same time, the biochemical response at the level of the main lipid classes in horse mussels under all the studied crude oil concentrations was in many respects similar to the response of blue mussels [24,25]. As was the case with the blue mussels [24], the Ch/PL ratio in the horse mussel gills decreased on the tenth day under all the studied crude oil concentrations, indicating an increased permeability of the biological membranes at the end of the experiment [12]. Similarly, the rise in the TAG levels on the third day of the experiment may point to the activation of the accumulation and detoxication processes through autophagy [24,35]. It is well known that neutral lipids or TAG are also sequestered by autophagy in the lysosomes of mussels exposed to PAHs [35]. As with the blue mussels [25], the increased Ch/PL ratio in the horse mussel digestive glands indicated a decrease in the permeability of the biological membranes on the third day of the experiment and on the first experimental day under exposure to the high crude oil concentration. At the same time, the reduction in the TAG levels in the digestive glands of the horse mussels probably reflects the utilization of the lipid pool in various biosynthetic processes including additional synthesis of the membrane lipids.

The main components of biological membranes are phospholipids, which are dominated by PC and PEA, whereas minor phospholipids (PS, PI, LPC, and SM) play an important regulatory role in many cellular processes [36]. Despite the increase in the PC concentration, there was an increase in the PEA/PC ratio against the background of the low Ch/PL ratio in the gills of the blue mussels and the horse mussels on the first and tenth days of the experiment, confirming the assumption about the increasing permeability of the biological membranes of the mussel gills in response to the different crude oil concentrations. It should be noted that the opposite effect was observed in the digestive glands on the third and tenth days of the experiment (i.e., PEA/PC ratio reduction), indicating a decrease in the permeability of the cell membranes in the digestive glands in response to crude oil pollution. A similar response in all studied bivalve groups acclimated to predominantly 25 psu salinity at the membrane lipid (PC, PEA, and cholesterol) level in response to different crude oil concentrations in seawater probably reflects the common adaptive biochemical mechanisms in the structural organization of cell membranes [12,13,14]. Apparently, the primary compensatory response of the lipid composition to crude oil exposure mainly on the first day of the experiment occurred in the mussels’ gills, the site of first contact with the external environment. In the digestive glands, the organ responsible for the detoxification of xenobiotics, the increased cholesterol content, and reduced PEA/PC ratio in the membranes (providing lower membrane permeability) after three days of crude oil exposure served as a biochemical adaptive mechanism of the mussels to the narcotic effect of crude oil [10,11]. By the end of the experiment, the mussels appeared to be acclimated to the new environmental conditions, and the membrane and storage lipid content and their ratios reached mostly new levels or returned to their initial values. The consistency of the data for the lipid composition modifications despite the variations in cardiac activity reflects the sensitivity of the physiological and biochemical indices in both horse mussels and blue mussels in response to crude oil exposure.

The increase in the PS content in the gills of all the investigated groups of mussels exposed to the low and medium crude oil concentrations over three days might have led to a modification in the activity of the enzymes, ion channels, and pumps involved in cell volume regulation [36]. However, an increase in the PI level was observed in the gills of the mussels exposed to the low and medium crude oil concentrations. PI plays an important role in essential metabolic processes. It is the precursor for diacylglycerols and phosphates such as IP3, which regulate the activity of protein kinase C [36]. Furthermore, PI is the main source of arachidonic 20:4n-6 acid, which is the metabolic precursor for the synthesis of eicosanoids, biologically active molecules [36]. Therefore, an increased level of the minor membrane phospholipids on the third day of crude oil exposure probably facilitated the activation of various membrane-bound regulatory processes for the mussels to adapt to the oil pollution.

LPC is also involved in the regulation of many cellular processes by modulating the activity of various regulatory proteins. It is formed by the action of various phospholipase A2 isoforms on PC and lecithin-cholesterol acyltransferase, which transfers a fatty acid from PC to cholesterol, forming cholesterol esters [36]. An elevated LPC level was observed only in the gills of the horse mussels exposed to the low and medium crude oil concentrations (after 10 days), and it was accompanied by increased ECh content, indicating a possible activation of the lecithin-cholesterol acyltransferase in response to oil pollution. The LPC content in the digestive glands in the horse mussels declined on the tenth experimental day, and in the blue mussels, it declined on the first day of the experiment. The decline was accompanied by modifications in the ECh level, depending on the crude oil dose and the duration of the exposure.

SM is not only a structural component of cell membranes that replaces PC in the case of its deficiency, but it is also a regulator of cholesterol synthesis [37]. SM biosynthesis metabolites participate as secondary messengers in many cellular processes as well as during adaptation to external stress factors [36]. Interactions between SM and Ch facilitate cholesterol homeostasis in cells by inhibiting the enzyme activity of the acyl-KoA cholesterol acyltransferase involved in cholesterol esterification and storing it in ester form. In the blue mussel digestive glands, the low SM content might have contributed to the reduction in cholesterol levels (at 15 psu salinity) and the increase in ECh levels (at 25 psu salinity). In contrast, there was an increase in SM and ECh levels in the gills of the blue mussels acclimated to 15 psu and the gills of the horse mussels on day 10 of the experiment.

### 4.3. Effect of Low Salinity and Oil Pollution on Lipid Composition in Blue Mussels

Changes in the levels of the main lipid classes in blue mussel gills and digestive glands point to higher energy costs and restructuring in the membranes to adapt to low seawater salinity (15 psu). Thus, the gills of mussels acclimated to 15 psu salinity had low levels of Ch, PS, PE, A and PC as well as a reduced PEA/PC ratio [24]. These features appear to provide for the regulation of gill cell membrane permeability to stabilize cell volume in hypo-osmotic environmental conditions. The high level of cholesterol and its esters as opposed to the lowered content of storage lipids (especially TAG) in the digestive glands of blue mussels indicates not only the high energy costs necessary for low salinity adaptation of the mussels, but also the cell membrane structure modifications [12,13,14]. In the blue mussels acclimated to 15 psu salinity, the response to crude oil pollution in the gills was characterized by an increase in cholesterol levels. At the same time, the digestive glands of the blue mussels acclimated to 15 psu showed an increase in the TAG level, as opposed to a decrease in the TAG level in the mussels at 25 psu. The lower cholesterol levels in the gills and the reduced TAG content in the digestive glands of the blue mussels acclimated to 15 psu appeared to augment the content of the lipid classes through their additional biosynthesis in response to the crude oil exposure. It can be inferred that when the Ch level in the total lipid content of gills and the TAG level in digestive glands reach a certain level, biochemical protective mechanisms are triggered to promote the mussels’ sustainability under exposure to crude oil. It should be noted that such modifications in the main lipid classes that occurred on the first day of crude oil exposure facilitated the adaptation of ‘low salinity’ mussels to crude oil pollution, as shown in the cardiac activity results.

## 5. Conclusions

In summary, the present study demonstrated a high sensitivity of cardiac activity and lipid composition in blue mussels and horse mussels to the presence of crude oil in seawater. This sensitivity manifested itself not only in changes in HR amplitude, but also in the response pattern of cardiac activity over the course of the experiment. Distinct HR variability (HR waves) was observed at all experimental concentrations of the oil products. Thus, mussel HR is a reasonable and promising biomonitoring instrument. This method allows for the conditions associated with pollution to be monitored in real time for as long as it is necessary. This is particularly important as a majority of the investigations of the effects of oil on the physiology/biochemistry of marine organisms are carried out after contamination has occurred. The modifications in the levels of the main membrane lipid classes (such as PC, PEA, and cholesterol) and storage lipids (primarily TAG) in response to different crude oil concentrations are similar in both of these bivalve species, which differ in sensitivity to environmental factors and represent a universal mechanism for the bivalves’ biochemical adaptation to crude oil contamination. The present study shows that lipid composition modifications in mussels depend on the concentration of the oil products and the duration of the exposure to them as well as the target organ. Moreover, the modification in the lipid composition of blue mussels in response to crude oil exposure is defined by the ambient salinity.

## Figures and Tables

**Table 1:** Nominal and measured total hydrocarbon content (THC, mg/L) in treatment waters at exposure start (initial) and following 1, 2, 3, and 10 days of exposure to low, medium, and high crude oil treatments. Values are mean ± standard error (*n* = 4).

Days	Low	Medium	High
0–Initial (nominal)	9	45	450
1 day (measured)	2.20 ± 0.64	3.50 ± 0.17	45.00 ± 3.80
2 days (measured)	0.80 ± 0.10	0.97 ± 0.04	18.60 ± 1.31
3 days (measured)	0.17 ± 0.04	0.29 ± 0.05	12.90 ± 2.03
10 days (measured)	0.05 ± 0.01	0.05 ± 0.01	4.80 ± 0.93

**Table 2:** Results of factorial ANOVA to determine the effects of treatment (control or different concentrations oil exposure) on the HR of the *M. edulis* and *M. modiolus* during the experiment (*p*).

Effects	1 Day	2 Days	3 Days	4 Days	5 Days	6 Days
Oil concentration	0.001	0.001	0.001	0.059	0.014	0.070
Species	0.001	0.060	0.001	0.001	0.001	0.001
Interaction	0.040	0.036	0.004	0.382	0.058	0.094

**Table 3:** Results of factorial ANOVA to determine the effects of treatment (control or different concentrations oil exposure) on the HR of the *M. edulis* under normal (25 psu) and low (15 psu) salinity during the experiment (*p*).

Effects	1 Day	2 Days	3 Days	4 Days
Oil concentration	0.006	0.001	0.001	0.527
Salinity	0.922	0.351	0.001	0.647
Interaction	0.768	0.006	0.001	0.224

## Data Availability

Data is contained within the article.
